# Activation of Retinoid X Receptor increases dopamine cell survival in models for Parkinson's disease

**DOI:** 10.1186/1471-2202-10-146

**Published:** 2009-12-11

**Authors:** Stina Friling, Maria Bergsland, Susanna Kjellander

**Affiliations:** 1 The Ludwig Institute for Cancer Research, Stockholm Branch, Box 240, S-171 77 Stockholm

## Abstract

**Background:**

Parkinson's disease (PD) is caused by degeneration of dopamine (DA) neurons in the ventral midbrain (vMB) and results in severely disturbed regulation of movement. The disease inflicts considerable suffering for the affected and their families. Today, the opportunities for pharmacological treatment are meager and new technologies are needed. Previous studies have indicated that activation of the nuclear receptor Retinoid X Receptor (RXR) provides trophic support for DA neurons. Detailed investigations of these neurotrophic effects have been hampered by the lack of readily available DA neurons *in vitro*. The aim of this study was to further describe the potential neurotrophic actions of RXR ligands and, for this and future purposes, develop a suitable *in vitro*-platform using mouse embryonic stem cells (mESCs).

**Results:**

We studied the potential neurotrophic effects of the RXR ligand LG100268 (LG268) and the RXR-Nurr1 ligand XCT0139508 (XCT) in neuronal cultures derived from rat primary vMB and mESCs. RXR ligands protect DA neurons from stress, such as that induced by the PD-modeling toxin 6-hydroxy dopamine (6-OHDA) and hypoxia, but not from stress induced by oxidative hydrogen peroxide (H_2_O_2_) or the excitotoxic agent kainic acid (KA). The neurotrophic effect is selective for DA neurons. DA neurons from rat primary vMB and mESCs behaved similarly, but the mESC-derived cultures contained a much higher fraction of DA cells and thus provided more accessible experimental conditions.

**Conclusions:**

RXR ligands rescue DA neurons from degeneration caused by the PD simulating 6-OHDA as well as hypoxia. Thus, RXR is a novel promising target for PD research. mESC-derived DA cells provide a valid and accessible *in vitro*-platform for studying PD inducing toxins and potential trophic agents.

## Background

PD is caused by progressive degeneration of dopaminergic neurons in the substantia nigra of the vMB [1-3]. The resulting lack of the neurotransmitter DA leads to decreased signaling within the nigro-striatal pathway and produces disturbed regulation of movement with tremor, bradykinesia and rigidity [4]. As of today, there is no cure for PD.

Nuclear hormone receptors (NRs) are emerging as interesting factors in PD research. Most NRs are regulated by small, lipophilic ligands that easily enter the cell nucleus to control transcription. RXR (NR2B1-3) is activated by the synthetic ligand LG268 [5]. Interestingly, this activation has been shown to rescue DA neurons from degeneration in survival assays based on primary cultures [6]. Increased survival was dependent on activation of the heterodimer between RXR and the orphan NR, Nurr1 (NR4A2), as substantiated by several findings. Survival was selective for Nurr1-expressing neurons in vMB as well as cortex and the effects were abolished in cortical cultures from Nurr1 knock-out mice. Furthermore, the ligand XCT, which is selective for the Nurr1-RXR heterodimer, also increased vMB DA neuron survival. Nurr1 is essential for vMB DA neuron development [7], regulates genes essential for DA synthesis and storage [8-10], and has been indicated to have a role in neuroprotection of mature DA cells in several studies [11,12]. Indeed, human Nurr1-mutations have been associated with familial PD [13], providing clinically relevant evidence for such a role.

The origin of DA cell degeneration in PD is largely unknown, but it is suggested to be caused by agents causing oxidative damage and energy depletion in the brain [14-16]. The hydroxylated DA analogue 6-OHDA is commonly used to model nigral degeneration in experimental animals as well as *in vitro*, where it causes DA cell death and neurotransmitter depletion [17]. *In vivo*, its uptake is selective for DA cells through the DA transporter, and when inside the neuron, 6-OHDA produces oxidative stress [18,19] as well as mitochondrial inhibition [20]. Several other stressors can also be used to induce neurodegeneration and stress, for example hypoxic environments [21], the oxidative agent H_2_O_2 _[22,23], and the excitotoxic glutamate analogue KA [24-26].

Primary neuronal cultures provide data of high biological relevance. However, their use is diminished by low yields and high technical demands. Recently, we developed a platform for *in vitro *studies using DA cells derived from mESCs. Overexpression of the homeobox domain containing transcription factor Lmx1a under the control of the neuroprogenitor specific *Nestin *enhancer (NesE) induces formation of high numbers of *bona fide *vMB DA neurons in culture [27]. These neurons express all relevant neurotransmitters, show proper electrophysiological characteristics as well as physiological levels of DA and metabolites. Moreover, they survive and regenerate when grafted into 6-OHDA lesioned rat brains [28].

Here we have used DA neurons derived from primary vMB cultures as well as mESCs to further establish the neurotrophic role of RXR activity. We can show that RXR ligands selectively protect DA neurons from stress caused by 6-OHDA and hypoxia, but not from KA and H_2_O_2_. The protective effects are only seen in Nurr1-expressing DA cells. To conclude, RXR ligands and mESC-derived DA cells represent promising platforms in the search for novel PD therapies.

## Results and discussion

Since primary neurons are retrieved directly from developing brain tissue, artifacts are small, assuring data of high biological relevance. Primary neuronal cultures were prepared from rat E14.5 vMB. The initial extensive neurodegeneration declined significantly within approximately 24 hours of plating and after three days *in vitro *(DIV) the cell death was negligible, whereby the cultures could be maintained for weeks (also see [29]). After three DIV, neurons have clearly visible neurites and a mature neuronal morphology. Approximately 2-3% of the neurons in the vMB cultures was dopaminergic and could be identified by immunostaining against the rate-limiting enzyme in DA synthesis, tyrosine hydroxylase (TH). To investigate the specificity of the RXR effects on neuronal survival, several stressors were used.

### RXR ligands rescue primary vMB DA neurons from 6-OHDA-induced degeneration

**Figure 1:**
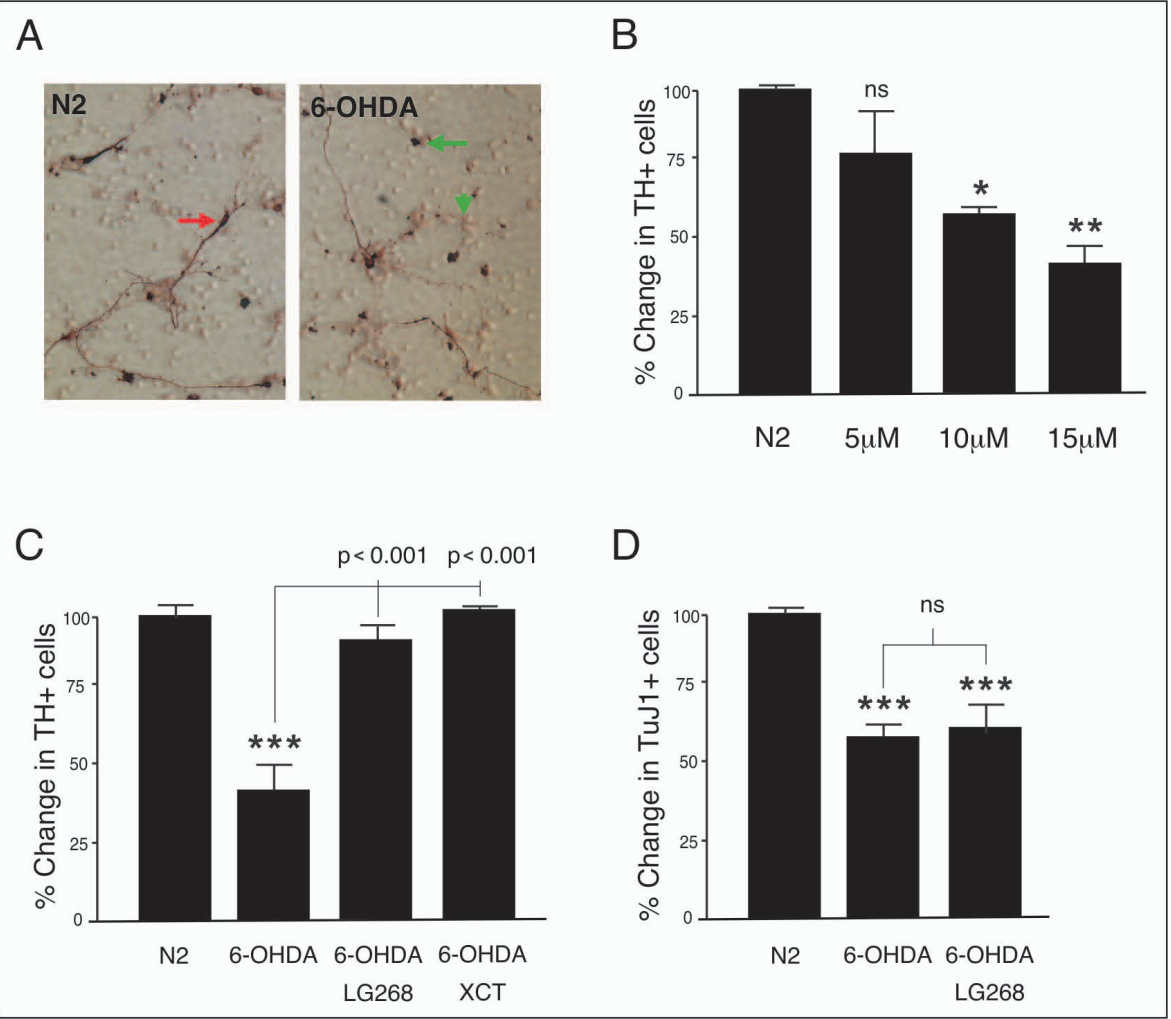
**RXR ligands selectively rescue primary DA neurons from 6-OHDA-induced toxicity**. A) Primary vMB cultures stained for TH-positive DA neurons (red arrow). 6-OHDA-exposure caused neurodegeneration, leaving cell debris (green arrow) and damaged neurites (green arrowhead). B) 6-OHDA produced a dose dependent toxicity of primary DA cells. 20 minutes exposure of 5, 10 and 15 μM 6-OHDA reduced the number of DA cells by 21%, 40% and 60%, respectively. C) In cultures stressed by 15 μM 6-OHDA, LG268 and the selective Nurr1-RXR ligand XCT rescued the DA neurons from degeneration. D) LG268 had no effects on the total amount of TuJ1-positive cells after 6-OHDA treatment. Stars (*) refer to differences between control-group (N2) and experimental-group, whereas values above brackets refer to differences between different experimental-groups. ns denotes non-significant, * denotes p < 0.05, ** denotes p < 0.01 and *** denotes p < 0.001 according to Tukey's multiple comparison test.

To simulate PD-like neurodegeneration, primary vMB cultures of three to five DIV were exposed to the neurotoxic DA analogue 6-OHDA. RXR ligands were added three hours prior to the toxin, and after 20 minutes of 6-OHDA exposure the medium and ligands were replaced to avoid unspecific stress from oxidized 6-OHDA. After additional 24 hours, cells were fixed, stained and scored. 6-OHDA produced clear signs of neurodegeneration in the TH-positive population, with evident neuronal loss, leaving cell debris and damaged neurites (Figure 1A). The degeneration was dose dependent (ANOVA, p < 0.01, F 12.6) (Figure 1B). 15 μM 6-OHDA reduced the number of DA cells by 60% compared to control (p < 0.01). Simultaneous exposure of the RXR ligand LG268, rescued the DA neurons from degeneration to 95% of control (p < 0.001, 6-OHDA vs. LG268 + 6-OHDA) (Figure 1C). RXR is ubiquitously expressed and it heterodimerizes with several NR subtypes. However, RXR-ligand dependent neurotrophic effects have been shown to be mediated through Nurr1 [6]. Here, the positive ligand effects after 6-OHDA exposure were only seen for the Nurr1 expressing DA neurons, whereas no neuronal rescue could be detected for the general neuronal population in the culture, as determined by scoring TuJ1-positive cells (Figure 1D). To further relate the trophic effects to Nurr1, we used the RXR ligand XCT, which is selective for the Nurr1-RXR heterodimer in the dose applied (1 μM, see [6]). Indeed, XCT rescued vMB DA neurons to 103% of control (p < 0.001, 6-OHDA vs. XCT + 6-OHDA) (Figure 1C). Without exposure to toxic stress, none of the ligands alone had any effect on cell number at three to five DIV (data not shown).

### RXR ligand rescues primary vMB DA neurons from degeneration caused by hypoxia, but not from KA and H_2_O_2_

**Figure 2:**
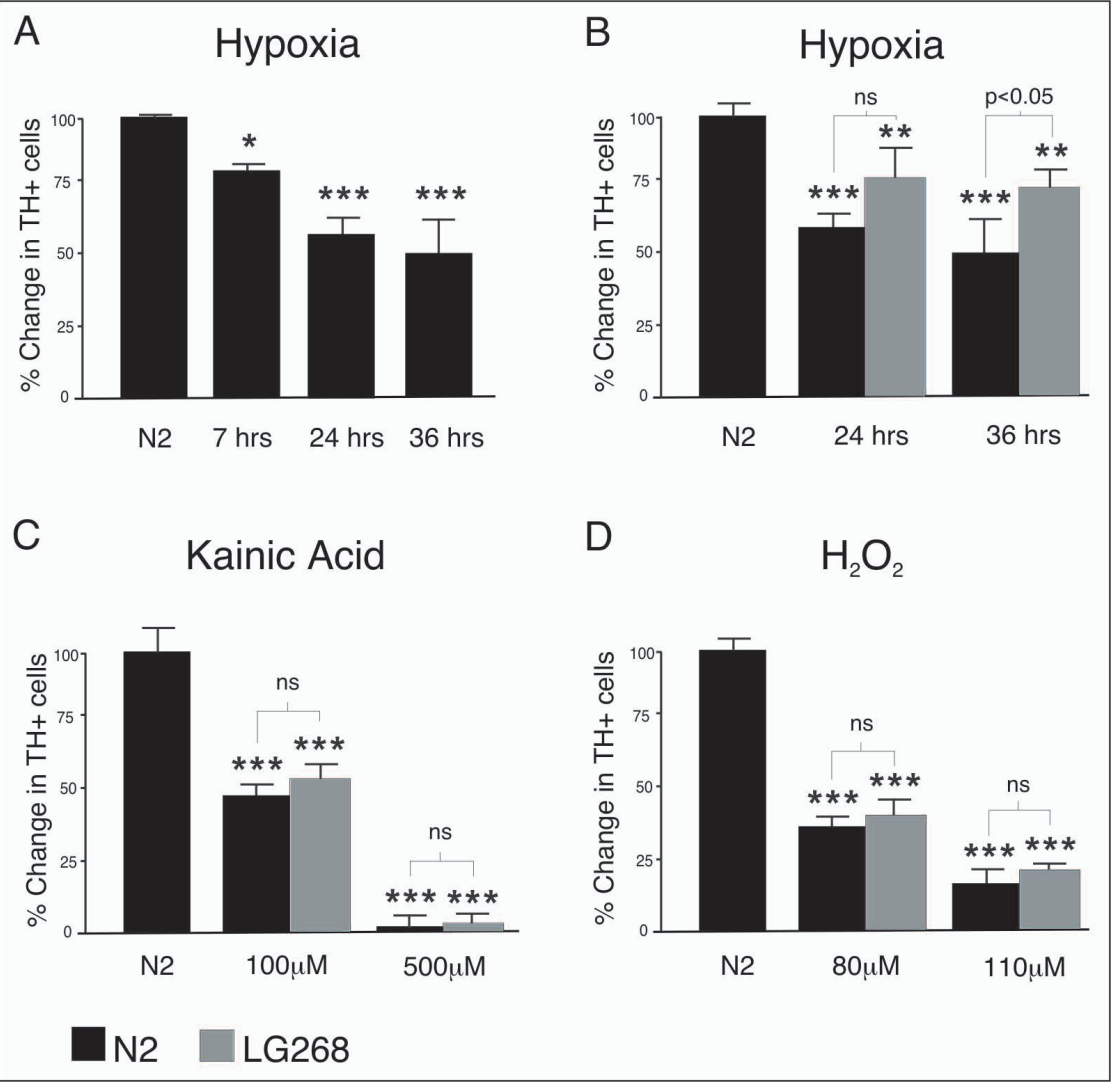
**RXR ligand rescues primary DA neurons from hypoxia, but not from KA or H_2_O_2_-induced toxicity**. A) Hypoxia induced a time dependent decrease in the number of primary DA cells, giving 18%, 42% and 49% reduction after 7, 24 and 36 hours in 0-1% O_2_, respectively. B) LG268 treatment (grey bars) during hypoxic conditions partly rescued the DA cells, giving a 30% and 40% increase in TH^+ ^cells compared to the cultures without added ligand after 24 and 36 hours, respectively. C) KA exposure at 100 and 500 μM reduced the number of primary DA cells by 52% and 98% compared to untreated cultures, respectively (black bars). LG268 provided no rescue (grey bars). D) H_2_O_2 _exposure at 80 and 110 μM gave 70 and 83% neurodegeneration, respectively (black bars). This could not be restored by addition of LG268 (grey bars). Stars (*) refer to differences between control-group (N2) and experimental-group, whereas values above brackets refer to differences between different experimental-groups. ns denotes non-significant, * denotes p < 0.05, ** denotes p < 0.01 and *** denotes p < 0.001 according to Tukey's multiple comparison test.

To study whether the neurotrophic activities of the RXR ligands were general or rather selective for specific cellular challenges, we studied their effects on several types of stressors and toxins. Primary vMB DA neurons of three to five DIV were stressed by hypoxic culture conditions (0-1% O_2_, 5% CO_2_). Ligands were added three hours prior to the hypoxic insult and kept throughout the experiment. Cells were fixed, stained and scored after different time intervals. In control culture conditions there is no decrease in the number of DA cells over time. However, hypoxia induced a time dependent decrease in DA cells (ANOVA, p < 0.001, F 55.2) (Figure 2A). Simultaneous exposure of LG268 during 24 and 36 hours in hypoxic conditions partly rescued the DA cells, giving a 30% (p = ns) and 40% (p < 0.05) increase, respectively, compared to the untreated cells (Figure 2B). Further, the excitotoxic glutamate analogue KA and the oxidative agent H_2_O_2 _were applied to primary vMB neurons of three to five DIV. KA as well as H_2_O_2 _exposure resulted in dose dependent degeneration of the DA neurons (KA, ANOVA, p < 0.001, F 277.9) (H_2_O_2_, ANOVA, p < 0.001, F 79.4) (Figures 2C and 2D). However, addition of the RXR ligands LG268 or XCT provided no neuronal rescue at any of the doses tested. This implies that the ligands are acting on specific cell death mechanisms, induced by 6-OHDA and hypoxia, but not on the calcium dependent excitotoxicity caused by KA or the pure oxidative stressor H_2_O_2_.

### RXR ligands rescue mESC-derived DA neurons from 6-OHDA-induced degeneration

**Figure 3:**
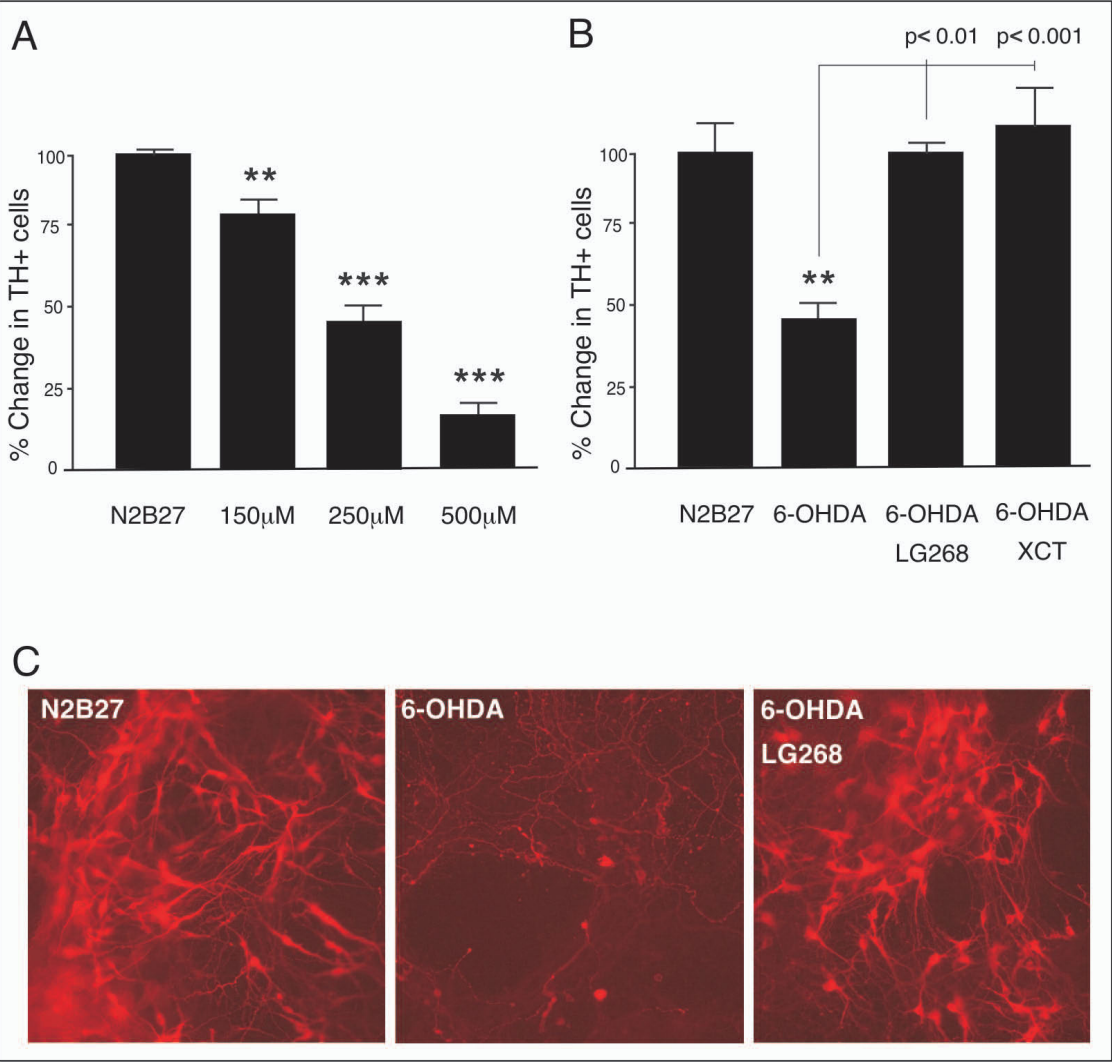
**RXR ligands selectively rescue mESC-derived DA neurons from 6-OHDA toxicity**. A) 6-OHDA produced a dose dependent toxicity in Lmx1a overexpressing mESC-derived DA cells. Three hours exposure of 150, 250 and 500 μM 6-OHDA reduced the number of DA cells by 33%, 55% and 82%, respectively. B) At 250 μM 6-OHDA, addition of LG268 and XCT completely rescued the DA neurons, from 55% reduction to 100% and 116% of control, respectively. C) Colony of mESC-derived DA cells stained red with Cy3-conjugated anti-TH antibody. 6-OHDA reduced the number of TH-positive cell bodies and remaining cells have degenerate morphology. Simultaneous treatment with LG268 rescued the TH-positive cells from degeneration. Stars (*) refer to differences between control-group (N2B27) and experimental-group, whereas values above brackets refer to differences between different experimental-groups. * denotes p < 0.05, ** denotes p < 0.01 and *** denotes p < 0.001 according to Tukey's multiple comparison test.

We further used mESC-derived DA cells as platforms for our evaluation of RXR ligands. These cells represent great progress for *in vitro *studies, since they provide a very high number of the correct cell type and homogeneous cultures. Stable NesE-Lmx1a mESCs (Lmx1a cells) were induced for neuronal differentiation as monolayer cultures in medium supplemented with the DA instructive cues Sonic Hedgehog (Shh) and Fibroblast growth factor (FGF) 8 [28]. After 18 days of differentiation, cells have a mature phenotype with long extended neuritis and expression of relevant DA cell proteins including TH (Figure 3, see also [28]). Cells were given RXR ligands for three hours and were then exposed to 6-OHDA for another three hours. Subsequently, media was changed and fresh ligands were added. Cultures were kept for additional 48 hours before fixation, staining and scoring. 6-OHDA produced a dose dependent toxicity, with reduced number of TH-positive cells and degenerate morphology (ANOVA, p < 0.001, F 83.9) (Figures 3A and 3C). As for the primary vMB neurons, simultaneous exposure of 6-OHDA stressed cultures to LG268 completely rescued the DA neurons from 55% (p < 0.01) reduction to 100% of control (p < 0.01, 6-OHDA vs. LG268 + 6-OHDA) (Figures 3B and 3C). No ligand-effect could be detected in the TuJ1^+^/TH^- ^neuronal population (data not shown). Also the selective Nurr1-RXR ligand XCT fully rescued the vMB DA neurons from 6-OHDA-induced degeneration (p < 0.001, 6-OHDA vs. XCT + 6-OHDA) (Figure 3B), suggesting an important role for Nurr1. Treatment of the cultures with RXR ligands without prior stressful insult did not affect the cell number (data not shown). Thus, the mESC-derived DA neurons reacted similar as primary neurons to the 6-OHDA stress and the RXR ligands. However, the much higher number of DA cells in culture substantially increased the power of the study. Furthermore, once the proper mESC line was established and the differentiation protocols were refined, the mESCs offered more accessible cultures than those of primary neurons, allowing faster and more experiments.

PD inflicts considerable suffering for the affected and their families, and it constitutes a great cost for the society. Today, the opportunities for pharmacological treatment are meager and mainly consist of substitution with the DA precursor Levodopa. However its medical potential decreases severely over time [30]. The development of neuroprotective or neuroregenerative therapies would provide great benefits. As such, glial cell line-derived neurotrophic factor (GDNF) has been tested in several clinical trials against PD [31-35]. Unfortunately, lack of efficacy and potential hazardous side effects has lessened the initial enthusiasm, and future development of GDNF-based drug therapies seems uncertain. Thus, there is a high need for alternative strategies to treat PD.

NR ligands are small and lipophilic, and they easily pass the blood-brain-barrier, providing excellent targets for the pharmaceutical industry. Indeed, the characterization of novel NR-mediated signaling pathways during the last decade has resulted in development of novel drugs used in the treatment of metabolic disease and cancer. Specifically, RXR ligands have limited toxicity in humans [36] and selective RXR ligands would minimize the risk of adverse effects. Our findings that RXR activity leads to neuroprotection [6] provide novel possible approaches to PD research. The neuroprotective effect is mediated, at least partly, by the Nurr1-RXR dimer. Here, we further show that RXR ligands provide neurotrophic support after stress induced by the PD modeling toxin 6-OHDA. The effects are selective for the Nurr1-expressing DA cells and do not affect total neuronal number in the cultures. Interestingly, neuroprotective effects are also seen after hypoxia-induced neurodegeneration. No protective effects are seen after excitotoxic or pure oxidative stress induced by KA and H_2_O_2_, respectively. Thus, the effects of ligand activation is not generally neuroprotective, but rather likely to affect individual cell death pathways.

## Conclusions

Ligands activating RXR and the RXR-Nurr1 heterodimer selectively protect DA neurons from stress induced by the PD-modeling toxin 6-OHDA and hypoxia. Thus, the regulation of RXR activity holds promises to contribute to a novel, alternative strategy in PD treatment. Furthermore, mESC-derived cultures offer accessible platforms of DA neurons and may provide novel means to conduct valid *in vitro*-based PD research.

## Methods

### Primary vMB Cultures

All experiments were performed in accordance with guidelines from the Swedish National Board for Laboratory Animals. Primary vMB cultures were obtained as previously described [6]. Briefly, vMB from rat embryos at stage E14.5 were dissected, mechanically dissociated and plated on poly-D-lysine coated 12 or 24 well plates in serum free medium (N2) consisting of a 1:1 mixture of MEM (Gibco, UK) with 15 mM Hepes buffer (Gibco, UK) and Ham's F12 medium (Gibco, UK). The mixture was supplemented with 6 mg/ml glucose, 1 mg/ml bovine serum albumine, 5 μg/ml insulin, 100 μg/ml transferrin, 60 μM putrescine, 20 nM progesterone, 30 nM selenium and 1 mM glutamine (Sigma). Cultures were incubated for three to five DIV before treatment. Cultures were kept in 37°C, 5% CO_2 _and 99% humidity unless otherwise stated.

### DA cells derived from Lmx1a-expressing mESCs

DA cells were derived from E14.1 mESCs as previously described [27,28]. Briefly, mESCs were propagated in feeder free conditions in DMEM (Invitrogen) supplemented with 2000 U/ml LIF (Chemicon), 9% KSR, 3% FBS, 0.1 mM non-essential amino acids, 1 mM pyruvate (Invitrogen) and 0.1 μM β_2_-mercaptoethanol (Sigma). For generation of stable ESC lines, 2 × 10^6 cells were nucleofected with 7 μg linearized NesE-mLmx1a-PGK-neo vector according to protocol (mouse ESC nucleofector kit, Amaxa biosystems Gmbh, Koeln, Germany), selected with G418, replated on gelatinized dishes and induced to differentiate in N2B27 differentiation medium [37] supplemented with 20 ng/ml bFGF, 100 ng/ml FGF8 and 70 nM Shh. Cells were incubated for 18 days before treatment. Cultures were kept in 37°C, 5% CO_2 _and 99% humidity throughout all experiments.

### Ligands and stressors

Experiments were performed in triplicates. The ligands (stock solutions in DMSO; LG268 and XCT, kindly provided by Dr. Mark Leibowitz at Ligand Pharmaceuticals and Dr. Peter Ordentlich at X-ceptor pharmaceuticals, respectively), were diluted to working dilutions in culture medium and added to the cultures for two to three hours prior to neurodegenerative stressors. Final concentrations were 0.1 and 1 μM for LG268 and XCT, respectively. The DA analogue 6-OHDA was given to the vMB primary neuron cultures at 5-15 μM for 20 minutes before medium and ligand replacement. Hypoxic stress was induced by placing cultures in a modular incubator chamber (Billups-Rothenberg Inc., Del Mar, CA) at 37°C filled with 5% CO_2 _and 0-1% O_2 _(balanced with N_2_) from one to 36 hours. The excitotoxic glutamate analogue KA and the oxidative agent H_2_O_2 _were added over night, in the range of 100 to 500 μM and 80 to 110 μM, respectively. To stress mESC-derived DA cells, 150 to 500 μM 6-OHDA in 0.1% ascorbic acid were added three hours before medium and ligand exchange. The cultures were left for another 36 hours in the incubator.

### Immunocytochemistry

Paraformaldehyde fixed cultures were incubated overnight with TH (1:1000, Pel-Freez, Arkansas), TuJ1 (1:1000, Babco) antiserum in PBS containing 5% fetal calf serum and 0.3% triton X-100. Following rinses, cultures were incubated with FiTC- and Cy3 conjugated secondary antibodies (Jackson, ImmunoResearch, West Grove, PA) for direct detection or with biotinylated secondary antibodies followed by detection of immuno-staining using the ABC immunoperoxidase kit from Vector (Buringame, CA).

### Microscopical analysis and image collection

Analysis, imaging and cell counting were performed on Eclipse E1000M and Eclipse TE300 microscopes (both Nikon) coupled to the Spot2 camera (Diagnostic Instruments, Sterling Heights, MI). Scoring was performed by cell counting, and counts were made blind to avoid observation bias. Statistical analyses were performed by one-way analysis of variance (ANOVA) followed by Tukey's multiple comparison test when appropriate.

## Abbreviations

6-OHDA: 6-hydroxy dopamine; ANOVA: one-way analysis of variance; DA: dopamine; DIV: days *in vitro*; FGF: Fibroblast growth factor; GDNF: glial cell line-derived neurotrophic factor; H_2_O_2_: hydrogen peroxide; KA: Kainic acid; LG268: LG100268; mESCs: mouse embryonic stem cells; NesE: *Nestin *Enhancer; ns: non-significant NRs: Nuclear hormone receptors; PD: Parkinson Disease; RXR: Rexinoid × receptor; Shh: Sonic Hedgehog; TH: tyrosine hydroxylase; vMB: ventral midbrain; XCT: XCT139508.

## Authors' contributions

SF constructed the stable Lmx1a mESC line, performed cell culture, experiments and analyses and participated in manuscript writing. MB participated in experiments and analyses. SK conceived of and designed the study, performed cell culture, experiments and analyses and drafted the manuscript. All authors read and approved the final manuscript.
